# CNARA: reliability assessment for genomic copy number profiles

**DOI:** 10.1186/s12864-016-3074-7

**Published:** 2016-10-12

**Authors:** Ni Ai, Haoyang Cai, Caius Solovan, Michael Baudis

**Affiliations:** 1 Institute of Molecular Life Sciences and Swiss Institute of Bioinformatics, University of Zurich, Winterthurerstrasse 190, Zurich, CH-8057 Switzerland; 2 Center of Growth, Metabolism and Aging, Key Laboratory of Bio-Resources and Eco-Environment, College of Life Sciences, Sichuan University, Chengdu, Sichuan, 610064 China; 3 Department of Dermatology, “Victor Babeş” University of Medicine and Pharmacy, Timisoara, Romania

**Keywords:** Copy number profile, CNA, Reliability assessment

## Abstract

**Background:**

DNA copy number profiles from microarray and sequencing experiments sometimes contain wave artefacts which may be introduced during sample preparation and cannot be removed completely by existing preprocessing methods. Besides, large derivative log ratio spread (DLRS) of the probes correlating with poor DNA quality is sometimes observed in genome screening experiments and may lead to unreliable copy number profiles. Depending on the extent of these artefacts and the resulting misidentification of copy number alterations/variations (CNA/CNV), it may be desirable to exclude such samples from analyses or to adapt the downstream data analysis strategy accordingly.

**Results:**

Here, we propose a method to distinguish reliable genomic copy number profiles from those containing heavy wave artefacts and/or large DLRS. We define four features that adequately summarize the copy number profiles for reliability assessment, and train a classifier on a dataset of 1522 copy number profiles from various microarray platforms. The method can be applied to predict the reliability of copy number profiles irrespective of the underlying microarray platform and may be adapted for those sequencing platforms from which copy number estimates could be computed as a piecewise constant signal. Further details can be found at https://github.com/baudisgroup/CNARA.

**Conclusions:**

We have developed a method for the assessment of genomic copy number profiling data, and suggest to apply the method in addition to and after other state-of-the-art noise correction and quality control procedures. CNARA could be instrumental in improving the assessment of data used for genomic data mining experiments and support the reliable functional attribution of copy number aberrations especially in cancer research.

**Electronic supplementary material:**

The online version of this article (doi:10.1186/s12864-016-3074-7) contains supplementary material, which is available to authorized users.

## Background

Since the introduction of molecular-cytogenetic technologies for whole genome copy number aberration screening [1, 2], considerable advances have been made to work with a variety of sub-optimal material (e.g. micro dissected samples, aspiration biopsies, paraffin embedded tissue), both in the areas of DNA preparation, labeling and platform technologies as well as in bioinformatic processing of the experimental read-out. However, DNA copy number profiles from current microarray and sequencing experiments sometimes suffer from the presence of systematical “wave patterns” [3] throughout the whole genome, where within each genomic segment the estimated copy number deviates from the true value which is supposed to be a constant. These wave artefacts disrupt the piecewise constant signal of the copy number data and may lead to false positives or negatives in identifying CNAs.

One of the known causes to the wave artefacts is differential DNA retrieval across chromosomal regions, which may be due to GC-content bias [4], DNA replication timing [5], differences in chromatin organization during DNA isolation [6] and damages to the DNA by fixation procedures [7]. Copy number profiles with heavy wave artefacts sometimes can be corrected if certain requirements are met. Marioni et al. developed a method to remove wave artefacts in copy number profiles for normal samples without obvious CNAs [8]. Wiel et al. suggested to eliminate waves in tumor profiles with many CNAs using calibration profiles [3]. Some methods correcting GC-content bias have also been implemented for microarray [4] and sequencing experiments [9]. However, the methods proposed in these studies have a limited ability to remove wave artefacts or put many restrictions on the type and variability of the input data itself. In addition to wave artefacts, large derivative log ratio spread (DLRS) [10] correlating with poor DNA quality also leads to unreliable copy number profiles.

The limited ability of existing experimental and bioinformatic methods to remove wave artefacts or to correct for source dependent DNA quality variations motivated us to devise a method for assessing if genomic copy number can be reliably estimated from a pre-processed, technology agnostic copy number profiling dataset. Rather than developing a method for improving the sample derived copy number profiles themselves, our primary intention here is to provide measures for the contamination of copy number profiles through artefacts and thereby to support decisions regarding the suitability of these copy number profiles for downstream data analysis and interpretation.

Zhang and Zhang previously designed such a measure in an explorative study [7] where they proposed using autocorrelation scanning profile (ASP) to evaluate data quality, and demonstrated on simulated data that the median of ASP (medASP) can be used as a discriminative metric. However, it will be shown in the “Discussion” section that medASP is not an adequate measure for real-world data of different scenarios.

In this paper, we assess the reliability of copy number profiles using a machine learning approach. From our experience and by experiment, we selected four features which are able to adequately represent the copy number profiles for reliability assessment, i.e. the number of steps that can be detected by the step-fitting algorithm in the copy number profile, a quantitative value indicating how much the copy number data is step-like, the number of segments induced by both CNAs and wave artefacts detected by circular binary segmentation [11, 12], and the mean of DLRS within segments. We will explain in detail about these features in the “Results and discussion” section and discribe the step-fitting algorithm by which the first two features are generated in the “Methods” section. Based on these features, we trained a classifier to predict the reliability of the copy number profile. We will describe the way of classifying reliable and unreliable profiles, and subsequently assigning them into one of the five subcategories each having a biological or experimental correspondence. Our reliability assessment method can also be adapted to assess copy number profiles generated by those sequencing platforms from which copy number estimates could be computed as a piecewise constant signal.

## Results

### Piecewise constant model and segmentation

Tumor samples often contain CNAs, in which chromosomal segments are found gained or lost in copy number, deviating from the normal diploid status. Genome-wide copy number can be depicted as a piecewise constant signal, where the change-points are the boundaries of the chromosomal segments that differ in copy number, and the constant value between a pair of change-points is the copy number of the corresponding segment. Copy number profiling by microarray and sequencing techniques gives noisy estimates of the true copy number at specific genomic positions, which can be modeled as follows:

Assume a series of *n* log ratio copy number estimates **x**={*x*_*i*_:*i*=1,2,⋯,*n*} ordered by genomic position. The piecewise constant model for the series is

1 $$ x_{i} = \mu_{i} + \varepsilon_{i}, \quad i=1,2,\cdots,n,  $$

where ***μ***={*μ*_*i*_:*i*=1,2,⋯,*n*} is a piecewise constant function and ***ε***={*ε*_*i*_:*i*=1,2,⋯,*n*} is a sequence of independent and identically distributed errors. Assuming a series of *m*+1 change-points ***τ***={*τ*_*j*_:*j*=0,1,2,⋯,*m*} where 1=*τ*_0_<*τ*_1_<⋯<*τ*_*m*_=*n*+1 delimit *m* segments with copy number level ***θ***={*θ*_*j*_:*j*=1,2,⋯,*m*} such that

2 $$ \mu_{i} = \theta_{j}, \quad i\in[\tau_{j-1},\tau_{j}), \quad j=1,2,\cdots,m.  $$

The errors are usually assumed to be Gaussian ***ε***∼*N*(0,*σ* ^2^) and supported by experimental data on self-self hybridizations [13], although this assumption is not crucial if the distances between successive *τ*_*j*_’s are large [14]. The DLRS is denoted by *σ* and estimated by the standard deviation of the error ***ε***.

Segmentation is applied to recover the genomic position of the boundaries and the underlying copy number for chromosomal segments from the noisy copy number estimates. Under the piecewise constant model, the segmentation problem is to find the change-points ***τ*** delimiting the segments and the copy number levels ***θ*** for each segment. If ***τ*** is known, *θ*_*j*_ can be estimated by the mean of the copy number estimates that fall in the *j*-th segment, that is

3 $$ \hat{\theta_{j}} = \frac{\sum_{i=\tau_{j-1}}^{\tau_{j}-1} x_{i}}{\tau_{j}-\tau_{j-1}}, \quad j=1,2,\cdots,m.  $$

Many segmentation algorithms have been proposed (see [15] and [16] for excellent review), among which the popular circular binary segmentation (CBS) algorithm [11, 12] was found to be one of the most accurate methods. Starting with the whole chromosome, the CBS algorithm detects change-points delimiting a sub-segment with a different copy number level in the middle of a larger segment, and does it recursively until no more change-points can be found in any of the segments. For any interval [*a*,*b*) and 1≤*a*<*b*≤*n*, let the null hypothesis be that the log ratio copy number estimates *x*_*a*_,*x*_*a*+1_,⋯,*x*_*b*_ are independent and identically distributed Gaussian and the alternative be that there is a sub-segment with different mean and same variance. CBS calculates the maximal *t*-statistic *T*=*max*_*a*≤*i*<*j*≤*b*_|*T*_*ij*_|,

4 $$ T_{ij} = \frac{\bar{Y}_{ij}-\bar{Z}_{ij}}{s_{ij}\sqrt{(j-i)^{-1}+(b-a+i-j+1)^{-1}}},  $$

where $\bar {Y}_{ij} = (x_{i+1}+\cdots +x_{j})/(j-i)$, $\bar {Z}_{ij} = (x_{a}+\cdots +x_{i}+x_{j+1}+\cdots +x_{b})/(b-a+i-j+1)$, and $s^{2}_{ij}$ is the corresponding mean squared error.

### Step detection and step-likeness quantification by step-fitting

Ideally, DNA is uniformly retrieved from the chromosomes during sample preparation, that is, the amount of DNA retrieved is proportional to the true copy number of the chromosomal segments present in the cells. In this case, the log ratio copy number estimates contain only abrupt jumps which satisfy the piecewise constant assumption. However, in practice, due to GC-content bias [4], DNA replication timing [5] and other biological phenomena such as differences in chromatin organization during DNA isolation [6] or damages to the DNA caused by formalin fixation [7], DNA is sometimes retrieved differentially across the genome, which adds artefacts in the form of waves to the otherwise piecewise constant signal. These wave artefacts adversely affect detecting change-points which truly delimit the gained and lost chromosomal segments.

Existing segmentation algorithms such as CBS are poorly suited to discriminate the change-points that are boundaries of the CNA segments from those introduced by wave artefacts. When the magnitude of the waves are less than that of the CNA signal, CBS leads to “hyper-segmentation”, in which many change-points caused by waves are detected in a single CNA segment. In the worst case where the true copy number signal is buried in the waves with comparable or even greater magnitude, change-points detected by CBS largely depend on the wave artefacts and are no longer boundaries of the CNA segments.

Fortunately, in practice, CNAs induce abrupt jumps which are visualized as steps and wave artefacts only induce gradual changes. As a result, for copy number profiles containing CNAs, reliable samples are usually step-like, with few waves in each CNA segment, whereas unreliable ones contain a lot of waves or the overall shape of the signal does not resemble steps any more. Based on these observations we implemented a fast step-fitting algorithm of time complexity *O*(*n*log*n*) adapted from the method originally proposed by Kerssemakers et al. (refer to Supplementary Methods 3 in [17]) to capture the step signal which are mostly CNAs regardless of the change-points introduced by wave artefacts, and assess how much the copy number profile is step-like. See “Methods” section for a detailed explanation about the step-fitting algorithm and the adaptation.

### Computer simulation: CBS versus the step-fitting algorithm

**Fig. 1:**
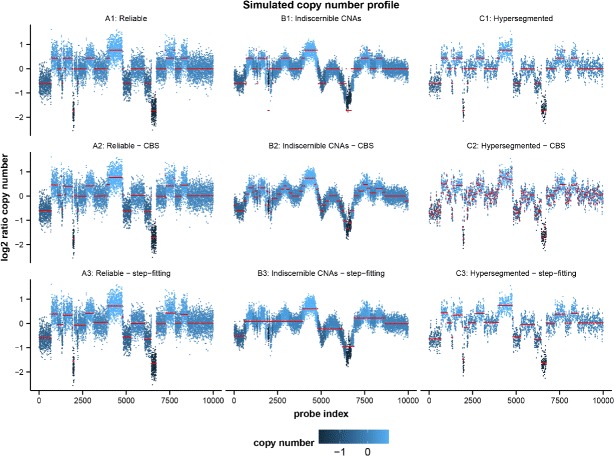
An example of simulated copy number profiles. Samples of 10,000 dimensions (*n*=10,000) were generated for 3 reliability groups, i.e. **A**: reliable copy number profiles containing many CNAs; **B**: unreliable copy number profiles having indiscernible CNAs due to wave artefacts; **C**: hyper-segmented copy number profiles. In group **A**, both CBS and step-fitting recovered majority of copy number segments well; In group **B**, both methods fitted noise and CBS detected more change-points than step-fitting; In group **C**, CBS detected too many change-points whereas step-fitting recovered majority of segments well

**Fig. 2:**
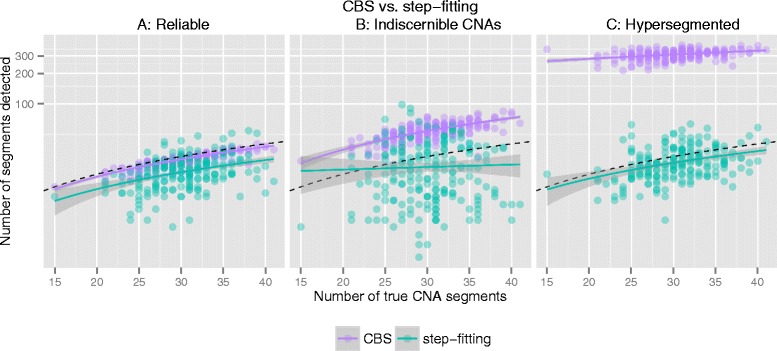
CBS versus step-fitting for 200 set of simulated copy number profiles. Scatter plot of the number of segments detected by CBS (*purple*) and step-fitting (*green*), plotted against the number of true CNA segments. In group **A**, the number of segments recovered by CBS approximates true CNA segments very well; the number of segments recovered by CBS is close to that of step-fitting. In group **B**, both methods fitted noise in which CBS found more change-points than step-fitting in general. In group **C**, CBS recovered much more segments than the number of true segments, whereas step-fitting found majority of CNA segments well

To demonstrate the differences between CBS and the step-fitting algorithm in detecting change-points, 3 groups of copy number profiles each containing 200 samples of 10,000 dimensions were generated (Figs. 1 and 2). Group A are reliable copy number profiles containing many CNAs; Group B are unreliable copy number profiles in which the piecewise constant CNA signal is buried in waves; Group C are hyper-segmented copy number profiles in which many change-points caused by waves are present in a single copy number segment. See Additional file 1 for details on how simulations were performed.

Figure 1 shows a set of simulated copy number profiles having the same true CNA segments. A1 to A3 demonstrate the segmentation on the same reliable copy number profile, where the red lines in A1 are the predefined true CNA segments. Red lines in A2 and A3 are copy number segments recovered by CBS and step-fitting respectively, from which it can be concluded that both methods recovered the majority of copy number segments. While CBS performs better in terms of detecting small segments of low signal to noise ratio, step-fitting is capable of capturing the overall step-like signal. B1 to B3 show the same unreliable copy number profile with heavy wave artefacts generated by introducing large auto-correlation to the reliable copy number profile in group A, for which neither CBS nor step-fitting can locate the true CNA segments correctly. In B2, CBS found greater amount of change-points most of which are noise, whereas in B3 step-fitting also fitted noise but only those with more abrupt changes and therefore the number of change-points detected are less than CBS; Nevertheless, change-points detected by both methods mainly depend on noise and are no longer boundaries of the true CNA segments. C1 to C3 show the same hyper-segmented copy number profile created by passing the reliable copy number profile in group A through a median filter to add small waves at the same time preserving the overall step-like structure. While in C2 CBS detected too many change-points within individual true CNA segments, step-fitting significantly outperformed by recovering majority of copy number segments well. Note here the possibility of merging multiple segments detected by CBS using additional methods is not considered, as our main goal is to find a good proxy for the extent of wave artefacts which is represented by the number of change-points that CBS can detect under its standard setting.

The observation from Fig. 1 can be generalized to the set of 200 simulated copy number profiles as shown in Fig. 2. For reliable copy number profiles in group A, the number of segments recovered by CBS is a very good approximation to the true CNA segments (Spearman correlation coefficient *ρ*=0.89; Regressing the number of recovered segments on the number of true segments by robust linear regression results in slope *w*=0.93 and intercept *b*=0.28), while step-fitting recovered majority of CNA segments well except for those very short segments of low signal to noise ratio (*ρ*=0.41; *w*=0.67 and *b*=0.79). For copy number profiles in group B containing heavy wave artefacts, both methods fitted noise. CBS recovered more segments than the number of true segments (*w*=1.86 and *b*=−2.26) and although in our simulation there is still correlation (*ρ*=0.70) as the waves introduced in different samples are of comparable size, the correlation is not expected in real data where the waves are of various size coming from different sources. Furthermore, in group B, the number of segments found by step-fitting is largely less than CBS and independent of the number of true segments (*ρ*=0.04; *w*=0.13 and *b*=19.56). Last, for hyper-segmented copy number profiles in group C, CBS recovered far more segments than the number of true segments (*ρ*=0.36; *w*=2.84 and *b*=222.80), whereas step-fitting found majority of CNA segments well (*ρ*=0.38, *w*=0.79 and *b*=2.21).

### Reliability assessment metrics

In this section we further develop the reliability assessment method on 1522 previously published copy number profiles from different experimental batches and microarray platforms (See “Methods” section for detail).

**Fig. 3:**
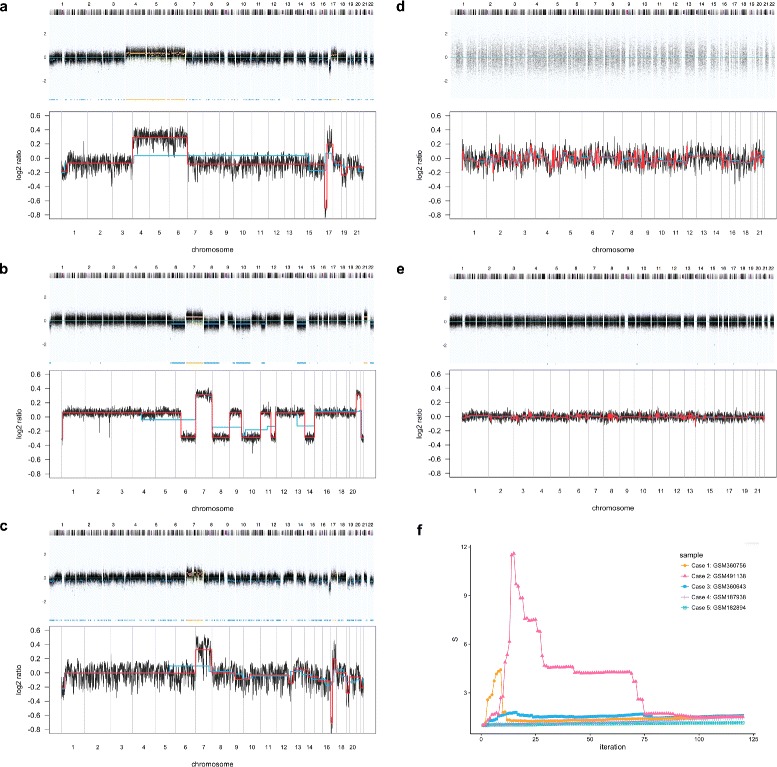
Five example specimen of copy number profiles for each of the reliability groups. 3**a**: *Case 1*, hyper-segmented, discernible CNAs with some waves; 3**b**: *Case 2*, reliable, discernible CNAs with few waves; 3**c**: *Case 3*, unreliable, indiscernible CNAs with heavy waves; 3**d**: *Case 4*, unreliable, large DLRS, undetectable CNAs; 3**e**: *Case 5*, reliable, control sample or without many CNAs. In each subgraph 3**a** to 3**e**, the *upper panel* shows the copy number profile segmented by the CBS algorithm, and the *lower panel* displays the same copy number profile segmented by step-fitting in the optimal iteration when *S*_*peak*_ was attained, where the red line is the fit and the blue line is the counter-fit. In Fig. 3 **f**, *S* values are shown for the same five copy number profiles. For each curve the *S*-values for 120 iterations are shown. The GEO accession numbers [29] for the five cases are: *Case 1*, GSM360756 [30]; *Case 2*, GSM491138 [31]; *Case 3*, GSM360643 [30]; *Case 4*, GSM187938 [32]; and *Case 5*, GSM182894 [33]

Figure 3 shows five typical cases each corresponding to one of the reliability groups that can often be observed among genomic copy number profiles. Apart from those containing many CNAs which can be classified as hyper-segmented, reliable and unreliable with heavy wave artefacts (Fig. 3 a to c), there exist copy number profiles that have no or very few CNAs (e.g. control samples from normal tissues; Fig. 3 d); but also copy number profiles with extraordinarily large DLRS suggesting poor DNA quality prohibiting the detection of any CNA (Fig. 3 e). The upper panel of each subgraph in Fig. 3 a to e shows copy number profile of a particular case segmented by CBS, and the lower panel shows the same copy number profile segmented by step-fitting in the optimal iteration when *S*_*peak*_, the maximum value of *S* generated by the step-fitting algorithm was attained. The corresponding *S* values throughout iterations are plotted in Fig. 3 f (See The step-fitting algorithm in “Methods” section for definition of *S* and *S*_*peak*_).

In Fig. 3 a to c, the performance of CBS versus step-fitting is consistent with that on simulated data in the previous section: In reliable copy number profiles (Fig. 3 b), segments recovered by CBS are comparable to those from step-fitting; in unreliable copy number profiles with heavy wave artefacts (Fig. 3 c) both methods fitted noise in which CBS found more change-points than step-fitting; in hyper-segmented copy number profiles (Fig. 3 a) CBS detected a great many change-points within individual steps recovered by step-fitting, resulting in a higher number of CBS derived segments. An additional feature associated with reliability can be discovered by looking closer into the three cases in which both the reliable and the hyper-segmented copy number profile have clear step-like structures, while in the unreliable case the boundaries of the CNA segments are much less defined. This property is reflected by the peak value of *S*: As shown in Fig. 3 f, the hyper-segmented (Case 1) and the reliable copy number profile (Case 2) both attained relatively high *S*_*peak*_ whereas *S*_*peak*_ for the unreliable copy number profile (Case 3) is much lower.

In Fig. 3 d and e, no apparent CNA exists or can be detected. The former is unreliable due to large DLRS so that the CNAs, even if are present, cannot be detected properly, whereas the latter is a reliable control sample diploid across the genome. Nevertheless, in both cases step-fitting merely fitted noise and the corresponding *S* values for Case 4 and 5 in Fig. 3 f increase throughout iterations yet remain close to 1 and no apparent peak can be observed.

So far we have discussed 4 features related to the reliability of copy number profiles, i.e. *S*_*peak*_, an indicator of how much the copy number profile is step-like; *l*, the number of steps detected by step-fitting; *v*, the number of segments detected by CBS which could be induced by both CNAs and wave artefacts; and *σ*, which is the DLRS estimated by the standard deviation of the error ***ε*** in Eq. (1).

The full set of reliability assessment metrics is summarized in Table 1, where *l* and *v* combined represent the wave density.

**Table 1:** Reliability assessment metrics for copy number profiles. Eight qualitative combinations lead to five reliability cases

Case No.	*S*_peak_	Wave density	*σ*	Assessment
		(*l* and *v*)		
1	high	high	high	hyper-segmented, discernible CNAs with some waves
	high	high	low	
2	high	low	high	reliable, discernible CNAs with few waves
	high	low	low	
3	low	high	high	unreliable, indiscernible CNAs with heavy waves
	low	high	low	
4	low	low	high	unreliable, large DLRS
5	low	low	low	reliable, control sample or without many CNAs

*S*_*peak*_ quantifies how much the copy number data is step-like; Wave density depends on two features *l* and *v*, where *l* is the number of steps recovered by step-fitting in the copy number profile and *v* is the number of change-points detected by CBS which could be induced by both CNAs and wave artefacts; And *σ* is the mean of DLRS within segments

### CNARA: reliability assessment for copy number profiles

To turn the qualitative metrics into quantitative ones and therefore allow to predict the reliability of a given copy number profile, a support vector machine (SVM) classifier was trained on the 1522 previously published copy number profiles labeled as reliable or unreliable by experts (see “Methods” section).

The 4 features *S*_*peak*_, *l*, *v* and *σ* were extracted for each of the 1522 samples and were used with the SVM classifier. The *svm* function in the *e1701* R package [18] which is the interface to the C++ implementation *libsvm* [19] was called, where the cost was set to 1 and the radical basis function (RBF) kernel *e* *x* *p*(−0.4|*u*−*v*|^2^) was chosen in which the parameters were set by cross validation. Tenfold cross-validation on the 1522 training samples resulted in a total prediction accuracy of 99.08 *%* with standard deviation 0.0083 (a 3D visualization can be found in Additional file 1: Figure S5). The extremely high accuracy suggests that the 4 features chosen can represent the copy number profile very well for reliability assessment. The resulted classifier can predict any new copy number profiles as reliable or unreliable in terms of whole-genome CNA evaluation. For fine tuning of the methodology to particular sample compositions or evaluation goals, we have included the option to use user provided training sets (see respective subsection below).

For a copy number profile predicted as reliable, the value of *S*_*peak*_ tells if it has many CNAs or not (case 2 and 5 in Table 1). Usually control samples from normal tissues or samples without many CNAs have *S*_*peak*_≤*t* *h* *r*_1_, while tumor samples containing CNAs that we are interested in have *S*_*peak*_>*t* *h* *r*_1_, where *t* *h* *r*_1_ is a threshold with default value 1.5.

Copy number profiles predicted as unreliable can be further divided into 3 subcategories (case 1, 3 and 4 in Table 1), by carrying out the following procedure: Denote samples in the training set by $(S_{peak}^{(i)},\ l^{(i)},\ v^{(i)},\ \sigma ^{(i)})$ where *i*=1, 2, ⋯, 1522. Given any unreliable sample *b*=(*S*_*peak*_, *l*, *v*, *σ*), create two dummy samples *d*_1_=(*S*_*peak*_, *l*, *m* *i* *n*(*v* ^(*i*)^), *σ*) and *d*_2_=(*S*_*peak*_, *l*, *v*, *m* *i* *n*(*σ* ^(*i*)^)) by substituting *v* or *σ* by the minimum of the training set correspondingly, and predict the reliability of *d*_1_ and *d*_2_. If *d*_1_ is reliable, *b* contains wave artefacts and therefore can be subcategorized as either Case 1 or 3; if *d*_2_ is reliable, *b* has large DLRS and thus belongs to Case 4; otherwise *b* suffers from both wave artefacts and large DLRS and can be either Case 1 or 3. As hyper-segmented samples in Case 1 may still be of interest for certain tasks such as looking for CNA regions but not counting the number or keeping track of the size of copy number gains or losses, a more stringent threshold *t* *h* *r*_2_ greater than *t* *h* *r*_1_ for *S*_*peak*_ could be set, for example, to accept those hyper-segmented samples having *S*_*peak*_>*t* *h* *r*_2_ with caution where the default value of *t* *h* *r*_2_ is 2.5.

## Discussion

### A comparison between CNARA and medASP

**Fig. 4:**
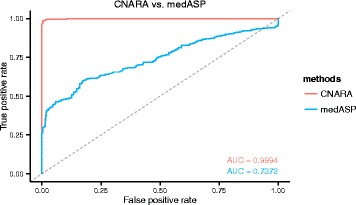
CNARA versus medASP. ROC plots of CNARA and medASP on the validation set. The 1522 samples were split into training and validation sets (50:50 %) at random, and the SVM classifier of CNARA was trained on the training set. The AUC of CNARA is 0.9994, compared to the AUC of medASP (0.7372)

The performance of CNARA and medASP [7] was compared and shown in Fig. 4. The 1522 samples were randomly split into training and validation sets at the proportion of 50:50 % and the SVM classifier of CNARA was trained on the training set where the parameters for the cost and the RBF kernel were set the same as in the previous subsection. The probability of being predicted as unreliable for each sample in the validation set was computed by the *libsvm* implementation [19] and the median of ASP was computed for the same sample in the validation set. The receiver operating characteristic (ROC) curves for the two values were then generated against the true class labels for the validation set.

As shown in Fig. 4, the area under the ROC curve (AUC) for medASP is 0.7372, which suggests that the assumptions made in the medASP study [7], i.e. simulating copy number profiles with a gain and a loss region of fixed size, does not comform well with feature distribution in real-world data. By contrast, the AUC of CNARA is 0.9994 which is consistent with the extremely high prediction accuracy of the SVM classifier stated in the previous subsection. The value approaching 1 once again implies that the 4 features chosen summarize the copy number profile very well in terms of its reliability.

### Custom training set

Apart from the training set consists of absolutely reliable and unreliable copy number profiles, CNARA is flexible to take in additional average quality samples labeled by experts to the training set to accomodate to their own need. For example, the boundaries of the CNA segments for formalin-fixed paraffin embedded (FFPE) samples are known to be less well defined than fresh-frozen samples in general [7, 20]. Therefore, when working with a large set of FFPE samples, one may wish to keep those slightly contaminated samples and reject others that are highly contaminated. To achieve this, people can create their own training set by including several slightly contaminated samples as reliable and highly contaminated as unreliable according to expert knowledge to the original training set (See Additional file 1 for more details on building custom training set).

## Conclusions

During our efforts on the curation of human cancer copy number data from genomic screening experiments [21, 22], we have frequently encountered data sets challenging the state-of-the-art quality assessment procedures. While several methods have been proposed to improve the readout of genomic copy number profiling both through improvements of experimental design [6] as well as application of advanced bioinformatic methods [3, 4, 8, 9], many data sets still contain artefacts that can not be removed sufficiently by these existing methods. As a result, when working with tens of thousands of genomic copy number profiles derived from a multitude of platforms and different pre-processing methods, a robust method capable of identifying the low quality data sets based on extractable features is needed.

Previously, some downstream quality control measures such as genotyping call rate [23] and derivative log-ratio spread (DLRS) [10] have been reported for microarrays to describe the noise induced during sample hybridization onto the arrays. However, existing evaluation methods are limited in controlling for inherent noise due to differential DNA retrieval across the genome during sample preparation, which is a severe problem in the generation of genomic copy number profiles, and therefore reliability assessment for copy number profiles remained an open problem. Zhang and Zhang previously did an explorative study [7] where they proposed medASP as a discriminative metric for evaluating the quality of copy number profiles and achieved good accuracy on simulated data. However, it has been shown in the previous section that medASP is not an adequate measure for real copy number data which contains different amount of copy number gains and losses of various sizes at different genomic positions.

In this paper, we proposed a method for assessing the reliability of DNA copy number profiles. We showed five typical cases each corresponding to one of the reliability groups that can often be observed in practice and discussed in detail about the 4 features which can represent the copy number profiles well in terms of reliability, namely the number of steps detected by step-fitting, an indicator of how much the copy number data is step-like, the number of segments induced by both CNAs and wave artefacts detected by CBS and the mean of DLRS within segments. To obtain the first two features we proposed a fast step-fitting algorithm of time complexity *O*(*n*log*n*) which is scalable to high-throughput copy number data. Taking these 4 features as input, an SVM classifer was trained on 1522 samples labeled as reliable or unreliable according to expert knowledge. By tenfold cross-validation on the whole dataset, the resulting classifier achieved a total accuracy of 99.08 % with standard deviation 0.0083. Predicted samples can be further subcategorized into the five reliablility classes, each having a biological or experimental correspondence. To the best of our knowledge, this is the first application developed for real-world data filling the gap of controlling the quality problem regarding differential DNA retrieval for copy number profiles, which is non-overlapping with and complementary to the objectives of any state-of-the-art quality control measures.

Our method can be applied to log ratio copy number data before or after normalization. Applied before normalization, it helps to judge if the log ratio copy number data needs normalization and noise correction; applied to the normalized data, it assesses the utility of the data in downstream analysis. Nonetheless, since it evaluates an aspect of the quality different from any existing quality control measures, we suggest our reliability assessment method to be the last step after any possible downstream quality control and bias correction methods, to decide if the normalized sample can be finally included in data mining experiments for biomedical knowledge generation. We have to emphasize that, while our method can deliver information about the quality of the signal derived from whole genome copy number screening experiments, by itself it does not address problems arising from the possible clonal heterogeneity, e.g. in biosamples derived from cancer tissues. Also, the impact of reliability assessment will depend on the intended downstream analyses; for instance, the reliability of whole-genome CNA profiles, as determined by our method, may be of less concern when using statistical CNA peak finding tools like GISTIC [24].

Thanks to the competing efforts on estimating genomic copy number from exome and whole-genome sequencing [25] especially recent development of CopywriteR [26] which is capable of extracting uniformly distributed copy number information from sequencing data, we are optimistic that in the near future piecewise constant signal for copy number estimates could be computed reliably with comparable acurracy to that of microarray platforms. Due to the universality of our method in dealing with samples from different platforms and the flexibility in taking new training samples, at that time our reliability assessment method can also be easily adapted to those copy number profiles generated by sequencing platforms.

## Methods

### The copy number profile dataset

1522 previously published copy number profiles (Additional file 2: Table S1) were used in our study. The samples were obtained from arrayMap [21, 22] with the original data retrieved from NCBI’s GEO repository [27], with preprocessing and noise correction performed through the standard arrayMap data processing pipeline [21]. Probe-level plots with added segmentation markers were visually inspected and selected by experts with respect to the empirically assessed copy-number calling reliability, and with the goal of a balanced representation of reliable and unreliable copy number profiles as well as a sufficient coverage of cases from different reliability groups. This selection resulted in 804 absolutely reliable copy number profiles (cf. cases 2 and 5 in Table 1) and 718 absolutely unreliable copy number profiles including hyper-segmented ones (cf. cases 1, 3 and 4 in Table 1). See Additional file 1 for data preprocessing and Additional file 2: Table S1 for a complete list of the arrays being analyzed and their reliability labels. The data is available at arraymap.org [21, 22].

### The step-fitting algorithm

To recover the set of change-points ***τ***={*τ*_*j*_:*j*=0,1,2,⋯,*m*} delimiting the CNA segments from the log ratio copy number estimates **x**={*x*_*i*_:*i*=1,2,⋯,*n*}, the algorithm updates ***τ*** iteratively, starting from ***τ*** ^(0)^={1,*n*+1}, in each iteration *k* introduces an additional change-point *τ* ^(*k*)^ to ***τ*** ^(*k*−1)^, called the best-fit, which minimizes the cost function

5 $$ H = \sum_{i=1}^{n}{(x_{i}-\mu_{i})^{2}}  $$

by scanning through all possible locations *i*=2,⋯,*n*, *i*∉***τ*** ^(*k*−1)^ and adding *i* to ***τ*** ^(*k*−1)^ as the temporary change-point set $\boldsymbol {\tau }_{temp}^{(k)}$ such that $\boldsymbol {\tau }_{temp}^{(k)} = \text {sort}(\boldsymbol {\tau }^{(k-1)} \cup i)$, in which the elements are ordered from the smallest to the largest; *μ*_*i*_ in Eq. (5) is computed from Eqs. (2) and (3) where $\{\tau _{j-1}, \tau _{j}\}\subseteq \boldsymbol {\tau }_{temp}^{(k)}$, *j*=1,2,⋯,*k*+1. The location *i* that minimizes Eq. (5) is therefore *τ* ^(*k*)^, and ***τ*** in the *k*th iteration is updated as ***τ*** ^(*k*)^=sort(***τ*** ^(*k*−1)^∪*τ* ^(*k*)^).

Next, in between each pair of *τ*_*j*−1_ and *τ*_*j*_ in ***τ*** ^(*k*)^ where *j*=1,2,⋯,*k*+1, find the change-point *c*_*j*_ which is the best-fit for *x*_*i*_, *i*∈[*τ*_*j*−1_,*τ*_*j*_) such that it minimizes

6 $$ F_{j} = \sum_{i=\tau_{j-1}}^{\tau_{j}-1}{(x_{i}-\psi_{i})^{2}},  $$

where *c*_*j*_∈(*τ*_*j*−1_,*τ*_*j*_) and

7 $$ \psi_{i} =\left\{ \begin{array}{ll} \sum_{i=\tau_{j-1}}^{c_{j}-1} x_{i}/(c_{j}-\tau_{j-1}) & \text{for}\ i\in[\tau_{j-1},c_{j})\\ \sum_{i=c_{j}}^{\tau_{j}-1} x_{i}/(\tau_{j}-c_{j}) & \text{for}\ i\in[c_{j},\tau_{j})\\ \end{array}\right.  $$

The set of change-points *c*_*j*_’s plus the boundary denoted by ***c*** ^(*k*)^={*c*_*j*_:*j*=1,2,⋯,*k*+1}∪{*c*_0_=1, *c*_*k*+2_=*n*+1} is called the counter-fit change-points for iteration *k*. The cost function *Q* for the counter-fit is defined as

8 $$ Q = \sum_{i=1}^{n}{(x_{i}-\nu_{i})^{2}}.  $$

where

9 $$ \nu_{i} = \frac{\sum_{i=c_{j-1}}^{c_{j}-1} x_{i}}{c_{j}-c_{j-1}}, \quad i\in[c_{j-1},c_{j}), \quad j=1,2,\cdots,k+2.  $$

The algorithm proceeds iteratively adding the best-fit change-point *τ* ^(*k*)^ each time to the change-point set ***τ*** ^(*k*−1)^ in the previous iteration and finding the set of counter-fit change-points ***c*** ^(*k*)^ correspondingly, until the number of iterations reaches a predefined threshold *K*. This results in a set of best-fit change-points and a set of counter-fit change-points located in between one another.

The step-fitting algorithm stated above (refer to Supplementary Methods 3 in [17] for more information) has time complexity of ∼2*n* *K* (for definition of the tilde notation see [28]) where *n* is the dimension of the copy number estimates **x** and *K* is the total number of predefined iterations usually greater than 100. An important observation which helps to improve the computational efficiency is that, of all the counter-fit change-points in ***c*** ^(*k*)^ in the *k*th iteration, the most prominent change-point *c*_*j*_ if added to ***τ*** ^(*k*)^ decreasing the cost function *H* the most is always the best-fit change-point *τ* ^(*k*+1)^ to be included in the next iteration, specifically,

10 $$ \tau^{(k+1)} = \underset{{c_{j}} \in \boldsymbol{c}^{(k)}}{\text{arg}\,\text{max}\,d_{j}}  $$

and

11 $$ d_{j} = \sum_{i=\tau_{j-1}}^{\tau_{j}-1}{(x_{i}-\mu_{i})^{2}-(x_{i}-\psi_{i})^{2}},  $$

where *i*∈[*τ*_*j*−1_,*τ*_*j*_), {*τ*_*j*−1_,*τ*_*j*_}⊆***τ*** ^(*k*)^, *c*_*j*_∈(*τ*_*j*−1_,*τ*_*j*_) and *j*=1,2,⋯,*k*+1; *μ*_*i*_ and *ψ*_*i*_ are computed as in Eqs. (2), (3) and (7) respectively. Here we keep track of the set of *d*_*j*_’s for each corresponding *c*_*j*_ as the difference set ***d*** ^(*k*)^. Furthermore, after the best-fit change-point *τ* ^(*k*+1)^ being included in ***τ*** ^(*k*+1)^, to update the corresponding counter-fit set ***c*** ^(*k*+1)^ and difference set ***d*** ^(*k*+1)^ we only need to exclude *τ* ^(*k*+1)^=*c*_*t*_ from ***c*** ^(*k*)^ and the corresponding *d*_*t*_ from ***d*** ^(*k*)^ first, and then scan the region delimited by the two best-fit break-points directly next to *τ* ^(*k*+1)^ in ***τ*** ^(*k*+1)^ for two additional counter-fit break-points and the corresponding two differences. To be specific, given *τ*_*r*_=*τ* ^(*k*+1)^, {*τ*_*r*−1_,*τ*_*r*_,*τ*_*r*+1_}⊆***τ*** ^(*k*+1)^, find *c*_*r*_∈[*τ*_*r*−1_,*τ*_*r*_) and *c*_*r*+1_∈[*τ*_*r*_,*τ*_*r*+1_) as computed in Eqs. (6) and (7), and compute *d*_*r*_ and *d*_*r*+1_ as in Eq. (11); then update ***c*** ^(*k*+1)^=(***c*** ^(*k*)^∖*τ* ^(*k*+1)^)∪*c*_*r*_∪*c*_*r*+1_ and ***d*** ^(*k*+1)^=(***d*** ^(*k*)^∖*d*_*t*_)∪*d*_*r*_∪*d*_*r*+1_. In this way, the time complexity is reduced to *O*(*n*log*n*).

To estimate the number of steps in the copy number profile and assess how much the data is step-like, the same model selection criterion is adopted as in Kerssemakers’ method, where the step-indicator *S* is introduced and defined as

12 $$ S=\frac{Q}{H}.  $$

For reliable copy number profiles or hyper-segmented profiles containing many steps which are mostly CNAs, in early iterations change-points in ***τ*** ^(*k*)^ and ***c*** ^(*k*)^ both locate significant steps such that *Q* is close to *H* and therefore *S* is close to 1. *S* increases until it peaks in the optimal iteration when change-points in ***τ*** ^(*k*)^ cover all significant steps and all change-points in ***c*** ^(*k*)^ merely fit noise so that *Q* and *H* differ the most. After that *S* decreases as change-points in both ***τ*** ^(*k*)^ and ***c*** ^(*k*)^ begin to fit noise and *Q* and *H* become closer again. As a result, the number of iterations it takes for *S* to reach the peak, denoted by *l*, is an estimation of the number of steps in the copy number profile.

In Kerssemakers’ literature they also mentioned the peak value of *S* denoted by *S*_*peak*_ approximates quadratic of signal to noise ratio given by $S_{peak} \approx 1+\frac {\Delta ^{2}}{4\sigma ^{2}}$, where *Δ* is the mean of the absolute difference of *μ*_*i*_’s around each best-fit change-point *τ*_*j*_ weighted by $\sqrt {\tau _{j+1}-\tau _{j-1}}$, where *τ*_*j*_∈***τ*** ^(*l*)^, *j*=1,2,⋯,*l*, and *σ* is the standard deviation of ***ε*** in Eq. (1). For unreliable copy number profiles of which the steps are drowned by wave artefacts of large magnitude, *S*_*peak*_ is significantly smaller than that of reliable copy number profiles containing comparable amount of CNAs. For those profiles containing few significant CNAs or control samples which are diploid across genome, *S* is close to 1 throughout iterations and no apparent peak can be observed so that *S*_*peak*_ is close to 1. Therefore *S*_*peak*_ is an indicator of how much the copy number profile is step-like.

The adapted step-fitting algorithm is summarized in Algorithm 1.



### Software

The CNARA software and a tutorial is available at https://github.com/baudisgroup/CNARA.

## Additional files

Additional file 1
**Supplementary Methods.** This document describes the computer simulation procedure for the 3 groups of copy number profiles in Figs. 1 and 2, the preprocessing procedure for the 1522 copy number profile dataset and the supporting **Figures S1-S5**, and the procedure of building custom training set. (PDF 933 kb)

Additional file 2
**Supplementary Table S1.** A complete list of the 1522 copy number profiles, including GEO accession number and reliability label. (TSV 58 kb)

## Abbreviations

ASP: Autocorrelation scanning profile
AUC: Area under the ROC curve
CBS: Circular binary segmentation
CNA/CNV: Copy number alterations/variations
CNARA: Reliability assessment for copy number profiles
DLRS: Derivative log ratio spread
FFPE: Formalin-fixed paraffin embedded
medASP: Median of ASP
RBF: Radical basis function
ROC: Receiver operating characteristic
SVM: Support vector machine
