# Supplementary material for: CNARA: reliability assessment for genomic copy number profiles
Source: BMC Genomics. 2016 Oct 12;17:799. doi: 10.1186/s12864-016-3074-7 (PMC5062840; doi:10.1186/s12864-016-3074-7)
Supplement: Additional file 1 — Supplementary Methods. This document describes the computer simulation procedure for the 3 groups of copy number profiles in Figs. 1 and 2, the preprocessing procedure for the 1522 copy number profile dataset and the supporting Figures S1-S5, and the procedure of building custom training set. (PDF 933 kb) [file 12864_2016_3074_MOESM1_ESM.pdf]

Supplementary
CNARA: reliability assessment for genomic copy number profiles

Ni Ai [1,*], Haoyang Cai [2], Caius Solovan [3] and Michael Baudis [1,*]

[1]Institute of Molecular Life Sciences, University of Zurich, Switzerland
[2]Center of Growth, Metabolism and Aging, Key Laboratory of Bio-Resources and Eco-Environment, College of Life Sciences, Sichuan University, Chengdu 610064, Sichuan, China
[3]Department of Dermatology, University of Medicine and Pharmacy "Victor Babes", Timisoara, Romania

[*]to whom correspondence should be addressed

Supplementary Methods

Computer simulation

In the simulation study comparing CBS with the step-fitting algorithm on change-point detection, 3 groups of copy number profiles each containing 200 samples of 10,000 dimensions were generated, in which Group A are reliable copy number profiles with well-defined CNAs; Group B are unreliable copy number profiles contaminated by heavy wave artefacts; Group C are hyper-segmented copy number profiles in which many change-points induced by wave artefacts are present in a single copy number segment. To make the simulation reflect the real data, each of the 200 samples for the 3 groups was generated as follows:

To take into account normal tissue contamination, assume each sample is composed of 30% of normal diploid cells and 70% of cancer cells containing CNAs of true copy number value $CN = (0, 1, 2, 3, 4, 5)$ ranging from full deletion to 5 copies for a particular segment. As a result, the log_2 copy number value each sample can attain is $CNValue = log_2((0.7 * CN + 0.3 * 2)/2)$.

generating true CNA segments

- Sample 50 boundaries from 9,998 positions (10,000 excluding the start and the end position).
- For each segment delimited by two successive boundaries, sample the corresponding copy number value $CNValue$ with probability weight $w = (0.02, 0.15, 0.61, 0.15, 0.05, 0.02)$, where the weight for diploid status is the largest, and decreases as the true copy number value CN gets further from 2.
- If two consecutive segments have the same $CNValue$, merge them into a single segment.

generating a sample for each of the 3 groups

Based on the true CNA segments generated,

- Group A (reliable): Add i.i.d. Gaussian noise $\boldsymbol{\varepsilon} \sim N(0, 0.09)$ to the true CNA segments to obtain x.

- Group B (indiscernible CNAs): Introduce large autocorrelation to x to obtain x_{wave} by adopting a similar strategy as in Zhang and Zhang [1].

$$n = 10,000,\ \beta = 0.7,\ lag = 150;$$
$$y = (x[lag+1],\ x[lag+2],\ \cdots,\ x[n],\ x[1],\ x[2],\ \cdots,\ x[lag]);$$
$$z = (x[n-lag+1],\ x[n-lag+2],\ \cdots,\ x[n],\ x[1],\ x[2],\ \cdots,\ x[n-lag]);$$
$$x_{wave} = x * (1-\beta) + (y+z) * \beta/2.$$

- Group C (hyper segmented): Add i.i.d. Gaussian noise $\boldsymbol{\varepsilon} \sim N(0, 0.36)$ to the true CNA segments and pass into a median filter of window size 10 to obtain $x_{hyperseg}$.

Data preprocessing of the copy number profile dataset

The data analyzed in the study consists of 1522 previously published copy number profiles retrieved from arrayMap [2, 3], for which preprocessing and noise correction was done by the project's data pipeline (as shown in Figure 5 of [2]) and the raw data is available at the Gene Expression Omnibus (GEO) [4] website. The total number of the copy number profiles were unknown beforehand. Samples were visually inspected and picked by experts, so that each sample was classified as "reliable" or "unreliable", while the amount of reliable and unreliable copy number profiles was balanced and cases from different reliability groups were well represented. This empirical selection process resulted in 804 äbsolutely reliablečopy number profiles (see ee.g. case 2 and 5 in Table 1 of the main article) and 718 äbsolutely unreliablečopy number profiles including hyper-segmented ones (cases 1, 3 and 4 in Table 1 of the main article). A complete list of the samples and the related information including platforms and reliability labels are given in Supplementary Table S1. Data input to the CBS and the step-fitting algorithm were then processed accordingly.

We observed that CBS tends to detect significantly more breakpoints for platforms of higher resolution such as the Affymetrix Genome-Wide Human SNP 6.0 Array (GEO GPL6801, purple, Figure S1). Thus to accommodate to platforms of different resolution, the first data point in every k_1 data points was kept where k_1 is the rounded value of the total number of data points in a sample divided by $100,000$. In this way, at most $100,000$ data points were kept for each sample and then fed into the CBS algorithm. As shown in Figure S1, the range of the number of change-points detected by CBS for down-sampled data is more consistent among platforms of different resolutions than the original data without down-sampling.

The step-fitting algorithm performed better on samples having greater signal-to-noise ratio (Figure S2). To reduce the noise, the copy number data was smoothed by a median filter of window size 100. To lessen the autocorrelation effect of the smoothed data without losing the entirety of the piecewise constant structure in the data, the first data point in every k_2 data points was kept for the smoothed data where k_2 is the rounded value of the total number of data points in a sample divided by $10,000$ (Figure S3). As a result, at most $10,000$ data points were kept for each sample; samples from different platforms could then be treated homogeneously by the step-fitting algorithm in downstream analysis.

The 4 features, i.e. S_{peak}, l, v and σ, as defined in the Reliability assessment metrics in the Results and discussion section of the main article, were then extracted for the preprocessed samples. The first 3 features were log-transformed to correct for skewness and then all 4 features were standardized (which means the resulted features have mean 0, standard deviation 1). The pairwise scatter plots of the 4 features are shown in Figure S4. A 3D visualization of the 4D features is shown in Figure S5, which demonstrates that the two classes (reliable/unreliable) represented by the 4 features are highly separable. Note that the principal component analysis (PCA) was adopted for the purpose of visualization only (it is a common way to visualize 4D in a 3D world where we are living); The input of the SVM is still 4 dimensional.

Building custom training set

We recommend that when building custom training set one should first consider adding their own training data into the dataset provided and retrain the model with all the data.

In the cases where this would not be suitable, given that our SVM classifier only uses 4 features we believe 100 samples should usually be enough to obtain a useful classifier. In practice, users should monitor this by splitting their data into a training set and a validation set, and train the model with subsets of different sizes of the training set, and track the prediction performance on the validation set. If for example, the performance on the validation set stops improving as soon as we are training with more than 50% of the data available, we would know that we have more than enough data to train the model. However, if the performance with 95% of the training data is a lot better than the performance with 85% then the model would almost certainly benefit from more training data.

References

1. Zhang, L., Zhang, L.: Use of autocorrelation scanning in DNA copy number analysis. Bioinformatics **29**(21), 2678–2682 (2013)

2. Cai, H., Kumar, N., Baudis, M.: arrayMap: a reference resource for genomic copy number imbalances in human malignancies. PLoS One **7**(5), 36944 (2012)

3. Cai, H., Gupta, S., Rath, P., Ai, N., Baudis, M.: arrayMap 2014: an updated cancer genome resource. Nucleic acids research, 1123 (2014)

4. Barrett, T., Wilhite, S.E., Ledoux, P., Evangelista, C., Kim, I.F., Tomashevsky, M., Marshall, K.A., Phillippy, K.H., Sherman, P.M., Holko, M., *et al.*: NCBI GEO: archive for functional genomics data sets–update. Nucleic acids research **41**(D1), 991–995 (2013)

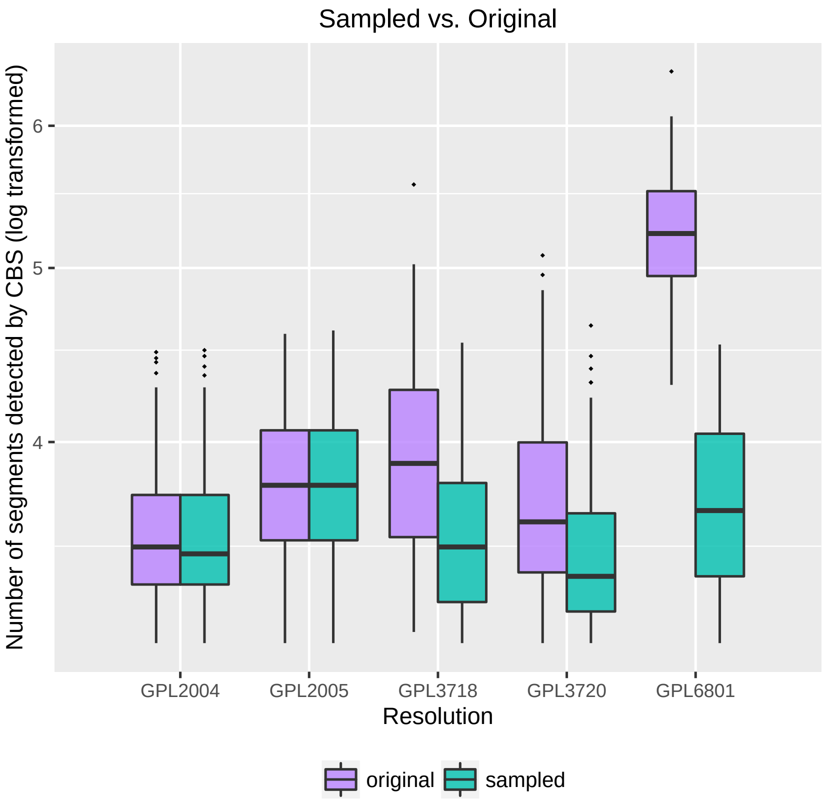

Figure S1: Boxplot of the number of change-points detected by CBS for sampled data (green) versus original data without sampling (purple) for platforms of different resolution. GPL2004 and GPL2005 have resolution of the order of 50,000; GPL3718 and GPL3720 have resolution of the order of 200,000; GPL6801 has resolution of the order of 1,800,000. The range of the number of change-points detected by CBS for sampled data are more consistent among platforms of different resolution than original data without sampling.

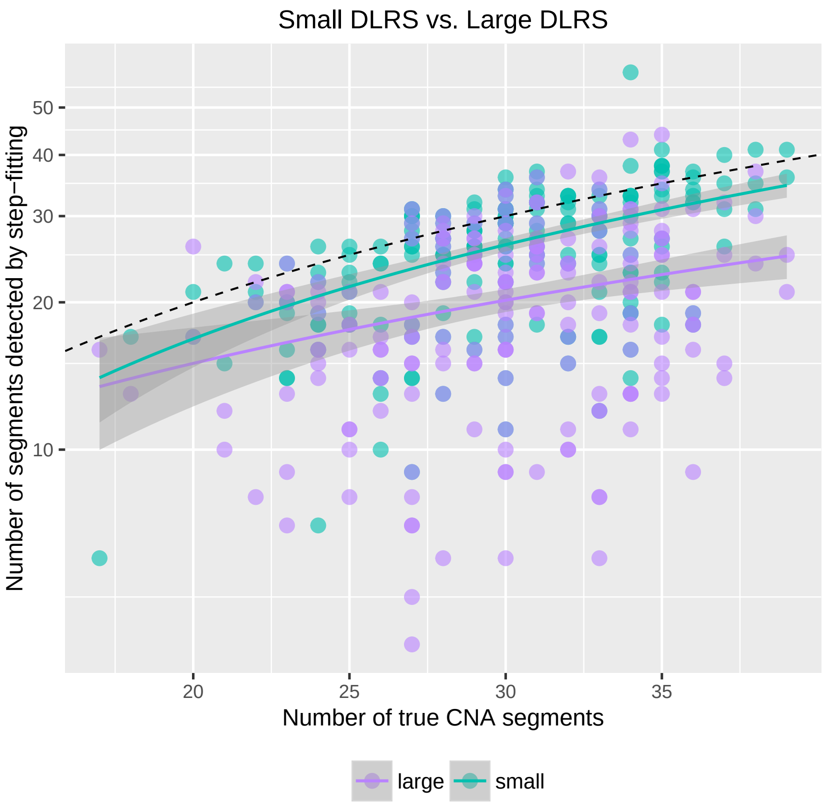

Figure S2: Simulation of 200 set of copy number profiles with small DLRS and large DLRS containing the same true CNA segments of 10,000 dimension. CBS on samples with small DLRS (green, Spearman's $\rho = 0.51$) outperforms that on large DLRS (purple, Spearman's $\rho = 0.29$).

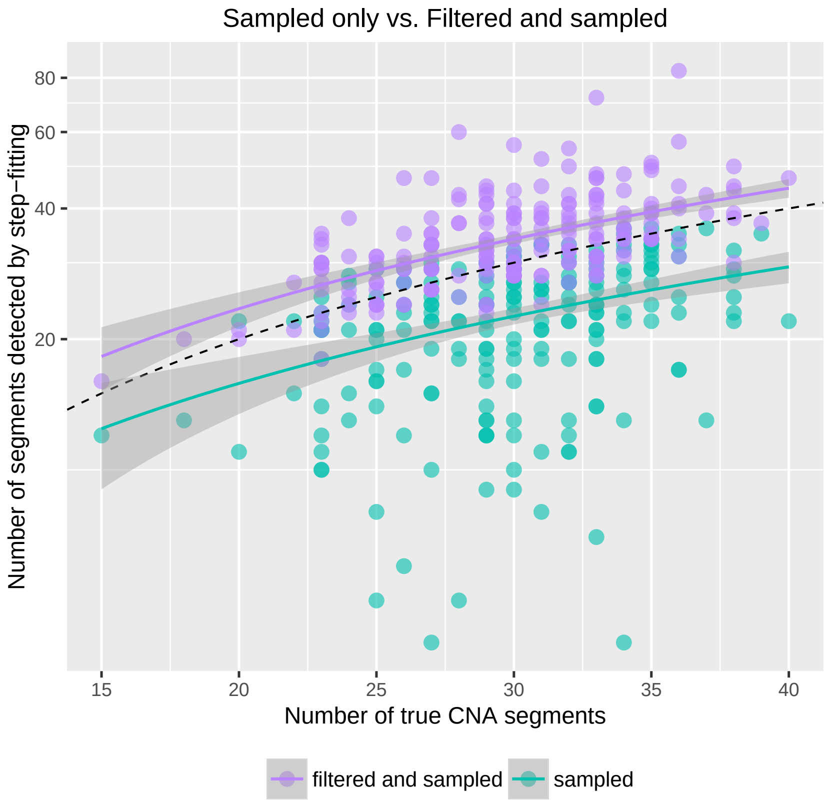

Figure S3: Simulation of 200 set of copy number profiles through different preprocessing procedure. The dimension of the samples is originally 200,000. Green shows the sampled only procedure and purple shows filtered and sampled, where purple (Spearman's $\rho = 0.59$) outperforms green (Spearman's $\rho = 0.41$). The resulted samples are of 10,000 dimension for both procedure.

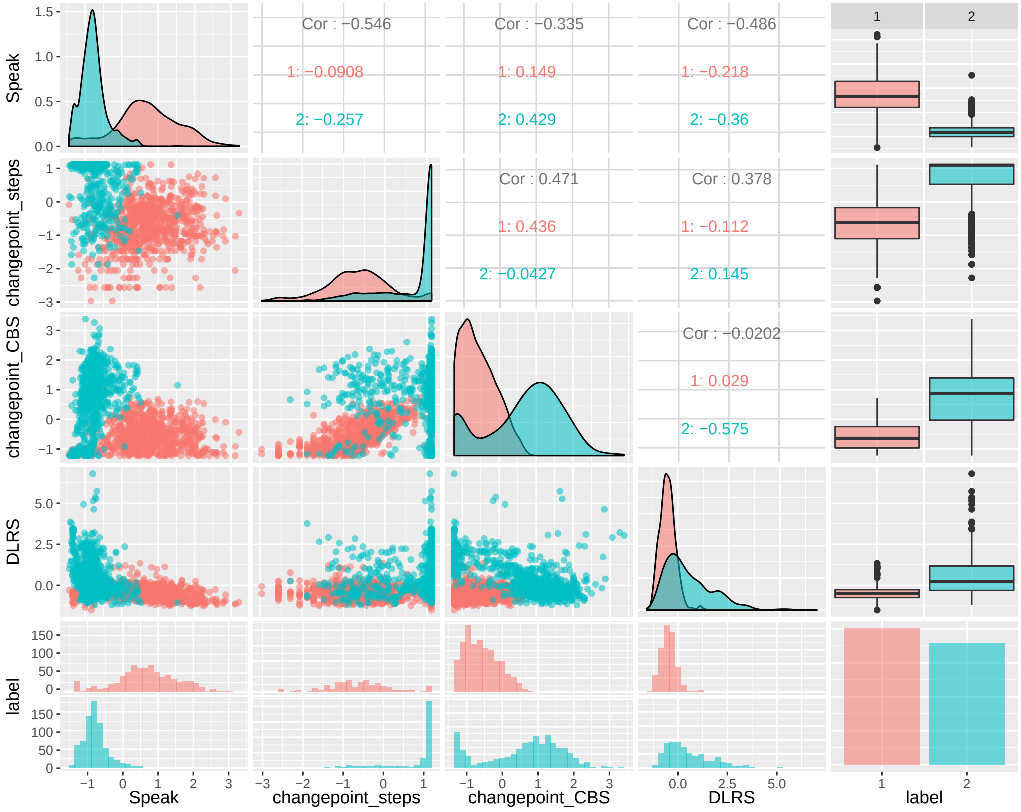

Figure S4: Pairs plot of the 4 features (log transformed and standardized). Red (label 1) denotes reliable; green (label 2) denotes unreliable.

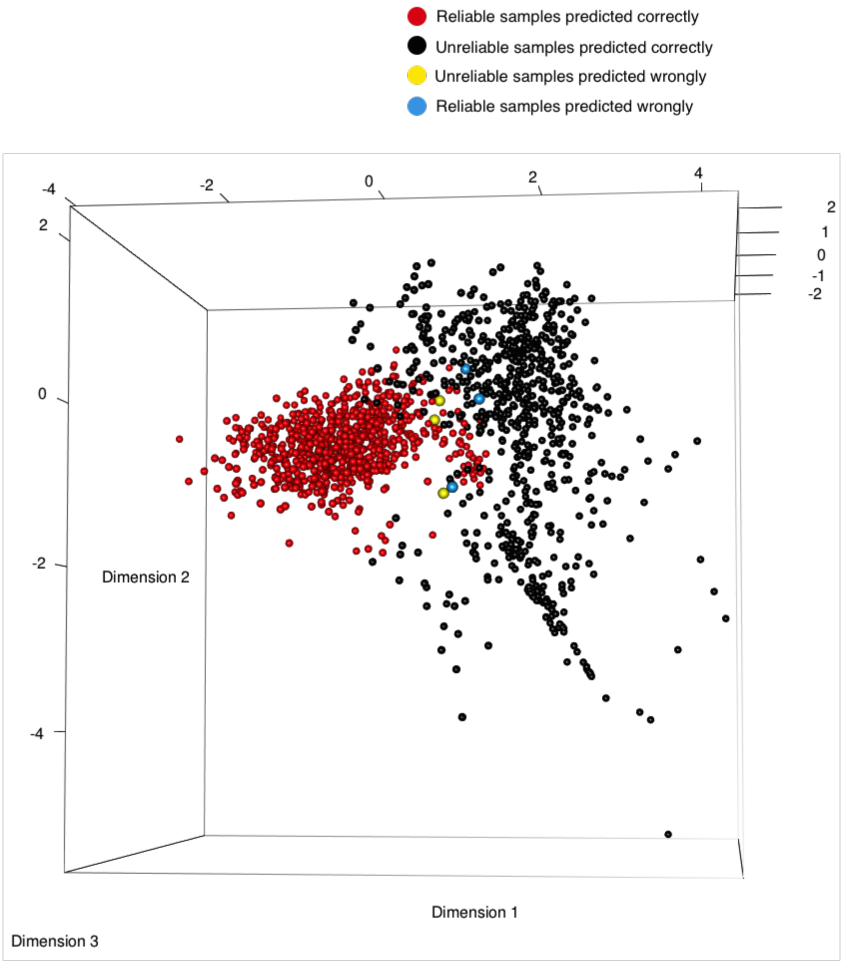

Figure S5: 3D visualization of the 4D dataset (1522 samples, Supplementary Table S1) where dimension reduction was achieved by principal component analysis (PCA) for the purpose of visualization only. Red are correctly predicted reliable samples; Black are correctly predicted unreliable samples; Yellow are unreliable samples predicted as reliable; Blue are reliable samples predicted as unreliable.
